# Monitoring Spatio-Temporal Changes of Terrestrial Ecosystem Soil Water Use Efficiency in Northeast China Using Time Series Remote Sensing Data

**DOI:** 10.3390/s19061481

**Published:** 2019-03-26

**Authors:** Hang Qi, Fang Huang, Huan Zhai

**Affiliations:** School of Geographical Sciences, Northeast Normal University, Renmin Street No. 5268, Changchun 130024, China; qih102@nenu.edu.cn (H.Q.); zhaih669@nenu.edu.cn (H.Z.)

**Keywords:** soil moisture, soil water use efficiency, land surface phenology, MODIS, northeast China

## Abstract

Soil water use efficiency (SWUE) was proposed as an effective proxy of ecosystem water use efficiency (WUE), which reflects the coupling of the carbon–water cycle and function of terrestrial ecosystems. The changes of ecosystem SWUE at the regional scale and their relationships with the environmental and biotic factors are yet to be adequately understood. Here, we aim to estimate SWUE over northeast China using time-series Moderate Resolution Imaging Spectroradiometer (MODIS) gross primary productivity data and European Space Agency climate change initiative (ESA CCI) soil moisture product during 2007–2015. The spatio-temporal variations in SWUE and their linkages to multiple factors, especially the phenological metrics, were investigated using trend and correlation analysis. The results showed that the spatial heterogeneity of ecosystem SWUE in northeast China was obvious. SWUE distribution varied among vegetation types, soil types, and elevation. Forests might produce higher photosynthetic productivity by utilizing unit soil moisture. The seasonal variations of SWUE were consistent with the vegetation growth cycle. Changes in normalized difference vegetation index (NDVI), land surface temperature, and precipitation exerted positive effects on SWUE variations. The earlier start (SOS) and later end (EOS) of the growing season would contribute to the increase in SWUE. Our results help complement the knowledge of SWUE variations and their driving forces.

## 1. Introduction

Soil moisture (SM) is usually expressed as a percentage of soil water content in dry soil weight. As one of the main driving forces of the water, energy, and carbon cycles in land surface and atmosphere [1], soil moisture plays an important role in promoting photosynthesis and ecosystem dynamics [2]. Water use efficiency (WUE) is defined as the ratio of gross primary productivity (GPP) to evapotranspiration (ET) of plants for the same period [3]. WUE is an indicator of the adjustment of vegetation photosynthesis to water loss [4], and quantitatively characterizing WUE can help us understand the interaction between the carbon and the water cycles of terrestrial ecosystems [5]. Soil moisture is affected by evapotranspiration, runoff, groundwater, etc. [6]. In arid grassland and shrubland, strong linear relationships were found between evapotranspiration and soil moisture [7]. During the late growing season, GPP is mainly affected by the soil moisture [8]. GPP is highly sensitive to the variation of surface soil moisture, especially in dry areas [9]. Soil moisture could be a good proxy of the rate of evapotranspiration at a large regional scale; therefore, it could be used to analyze the WUE of an ecosystem. Soil moisture use efficiency (SWUE), defined as the ratio between GPP and SM, was proposed as a new measure of WUE [9]. SWUE may depict the rate of carbon assimilation rate per unit of soil water consumption and reflect the interaction between soil water and ecosystem productivity [6]. A deeper understanding of how SWUE varies and its response to environmental and biotic factors is critical to accurately forecast terrestrial carbon and water cycles and feedback to climate change.

Traditionally, soil moisture data can be obtained via in situ measurement, which hardly meets the demands for continuous, large-scale monitoring. Due to the lack of understanding of the feedback mechanism of the atmosphere and insufficient explanation of the model errors, the retrieval of soil moisture data based on modeling may have a great impact on related research [10]. Satellite remote sensing in the microwave range constitutes a powerful source of information for monitoring large-scale variability of biomass, soil and vegetation moisture, and soil water use efficiency. Brandt et al. used the vegetation optical depth (VOD) derived from the Soil Moisture and Ocean Salinity (SMOS) mission to quantify inter-annual changes of aboveground carbon stocks in sub-Saharan Africa [11]. Fan et al. found good consistency between live fuel moisture content (LFMC) and the root-zone soil moisture (ECV_RZSM) derived from ECV_SM (the Essential Climate Variable near-surface soil moisture), the microwave polarization difference index (MPDI), and VOD in the south-eastern region of France [12]. The climate change initiative (CCI) project released by the European Space Agency (ESA) provides global daily soil moisture (SM) data to capture changes in precipitation and soil moisture models [13]. Dorigo et al. evaluated the ECV_SM products from 1988 to 2010 and found that the trends of ECV_SM products were consistent with other products [13]. The merged and individual soil moisture products (ECV_SM) were assessed using ground-based soil moisture data from 596 global stations during 1979–2010. It was found that ECV_SM merged products performed better than individual products except for ASCAT (Advanced Scatterometer) products and the quality improved over time [14]. He et al. used the CCI soil moisture and Moderate Resolution Imaging Spectroradiometer (MODIS) GPP to evaluate the variations in SWUE among biomes, climate conditions, and latitudes from 2000 to 2014 at a global scale [9]. 

Most existing studies on soil water use efficiency focused on crops at the field scale [15,16]. To our knowledge, the analysis of the relationships of large-scale SWUE and geographical, environmental factors, including elevation, soil types, vegetation types, and land surface temperature, is still unavailable. Land surface phenology (LSP) is the periodic plant life cycle, which evaluates the vegetation activity during the growing season at the ecosystem level [17]. Phenology directly or indirectly regulates carbon (e.g., photosynthesis and respiration) and water (e.g., transpiration and evaporation) fluxes between land surface and atmosphere from the plant to ecosystem scale [18]. The linkage of phenology to ecosystem SWUE remains to be investigated.

Northeast China is a typical region sensitive to global climate change, with abundant forests and grassland resources [19]. The carbon cycle of the terrestrial ecosystem in northeast China has great influence on regional climate, carbon budget, and the evolution of terrestrial ecosystems in the nation. Liu et al. studied the response of natural vegetation WUE to drought in northeast China [20]. Qiu found that WUE was mainly affected by temperature and precipitation [21]. Some existing research attempted to analyze land surface phenology variations and the effects of climate change in northeast China at different spatial and temporal scales [22,23,24,25]. However, no previous studies investigated the spatio-temporal patterns of soil water use efficiency and their impact factors in northeast China. 

The objectives of this study were to (1) examine the spatial and temporal patterns of ecosystem soil water use efficiency in the growing season (from April to October) across northeast China during 2007–2015; (2) evaluate the effects of vegetation cover, land surface temperature, and precipitation on soil water use efficiency changes; and (3) explore the responses of ecosystem soil water use efficiency changes to land surface phenology variation. This study helps improve the understanding of variabilities of soil water use in different ecosystems and their ecological function.

## 2. Data and Methods 

### 2.1. Study Area 

**Figure 1:**
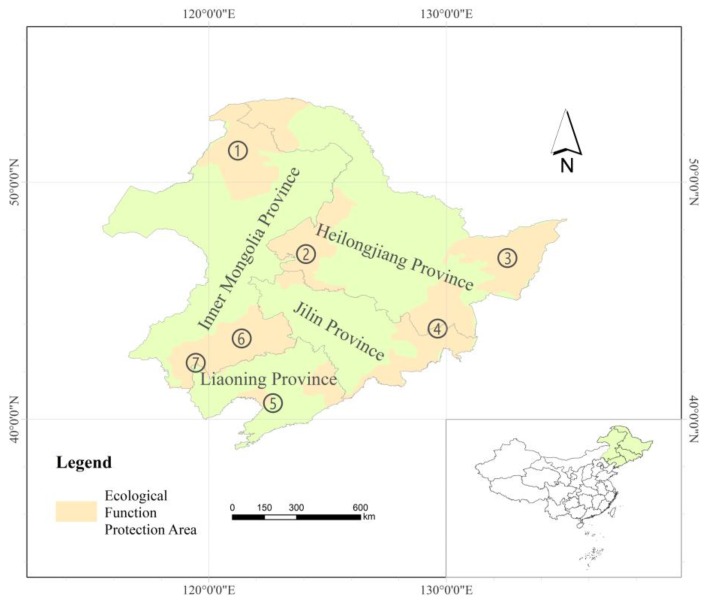
The geographical location of northeast China.

Northeast China was chosen as the study area, located between 38° north (N) and 53° N and between 115° east (E) and 135° E, covering an area of about 1,240,000 km^2^ (Figure 1). It includes Heilongjiang Province, Jilin Province, Liaoning Province, and the east of the Inner Mongolia Autonomous Region. Northeast China belongs to a temperate continental monsoon climate, characterized by four distinct seasons with short, warm summers and long, cold winters. The precipitation is mainly concentrated in the summer, and the annual precipitation decreases from southeast to northwest. The study area is surrounded by mountain ranges along three directions, including the Greater Khingan Mountains in the northwest, the Lesser Khingan Mountains in the northeast, and the Changbai Mountains in the southeast. The Liao River Plain, Songnen Plain, and Sanjiang Plain are located in the central and southern parts and the northeastern corner, respectively. The Hulun Buir Plateau is located in the western tip of the region. There are hills and tablelands situated between mountains and plains. Phaeozem is the main type of soil. The main vegetation types include deciduous broadleaf forests, deciduous coniferous forests, coniferous, and broadleaf mixed forests and grassland. Throughout the past few decades, northeast China suffered from eco-environmental problems, such as serious soil erosion, decrease of available cropland, serious flood disaster, deterioration of ecological environment, and decrease of grain yield and quality [26]. Several ecological function protection areas were established in the study area, including (1) the Greater Khingan Mountains (GKM), (2) Songnen Plain Wetland (SNPW), (3) Sanjiang Plain Wetland (SJPW), (4) the Changbai Mountains (CM), (5) Liaohe River Delta Wetland (LRDW), (6) Horqin Sandy Land (HSL), and (7) the Source Region of West Liao River (WLR). Monitoring and evaluating the changes of ecosystem SWUE under different hydro-climatic conditions in northeast China is significant to understand the response of ecological function to climate change.

### 2.2. Data Collection and Processing

#### 2.2.1. CCI Soil Moisture Product

CCI soil moisture product data were the basis of this study. The CCI project was a part of the ESA global essential climate variable monitoring project. The product consists of three surface soil moisture data sets: active products, passive products, and combined products. The active and passive soil moisture products were derived from two microwave scattermeters (ASCAT and European Remote Sensing (ERS) Active Microwave Instrument (AMI)) and four microwave radiometers (Tropical Rainfall Measuring Mission Microwave imager (TMI), Scanning Multichannel Microwave Radiometer (SMMR), Special Sensor Microwave Imager (SSM/I), and Advanced Microwave Scanning Radiometer-Earth Observing System (AMSR-E)), respectively. The combined products were merged by the first two datasets [27]. The spatial resolution of this daily soil moisture data was 0.25° with a reference time of 12:00 a.m. UTC (Coordinated Universal Time). These products measure the soil moisture at 0.5–2-cm depth. The soil moisture data of passive and combined products are in volume units (m^3^∙m^−3^), while the active products are expressed as a percentage of saturation (%) [28]. In this study, daily soil moisture data from April to October for the period of 2007–2015 were accessed at https://www.esa-soilmoisture-cci.org/. Then, eight-day soil moisture time series were generated using the maximum value composite (MVC) method. The images were subsets of the study area in ArcGIS.

#### 2.2.2. MODIS Time-Series Data

We selected MODIS products from 2007 to 2015 as the other main source of data, which were obtained from the level 1 and Atmosphere Archive and Distribution System (LAADS) Distributed Active Archive Center (DAAC), located in the Goddard Space Flight Center in Greenbelt, Maryland (https://ladsweb.modaps.eosdis.nasa.gov). The MODIS land surface temperature (LST) product (MOD11A2) provides daytime temperature with a spatial resolution of 1 km and temporal resolution of eight days [29]. We used the red and near-infrared reflectance bands of MOD09A1 product with a spatial resolution of 500 m to obtain eight-day NDVI (Normalized Difference Vegetation Index) time series [30]. NDVI data with a time resolution of 16 days were obtained from MOD13A2 with a spatial resolution of 1 km [31]. Datasets from MODIS eight-day GPP (MOD17A2H) at 500-m spatial resolution were collected [32]. The bilinear interpolation method was used to resample the MODIS data to a 0.25° resolution, which was consistent with that of SM data.

#### 2.2.3. Other Data

The elevation data were derived from SRTMDEM 90M product, using the Geospatial Data Cloud (http://www.gscloud.cn/). Shuttle Radar Topography Mission (SRTM) was measured by the National Aeronautics and Space Administration (NASA) and National Imagery and Mapping Agency (NIMA) [33]. The digital maps including ecological function protection areas, vegetation types, soil types, and ecogeographic zoning of China were downloaded from the Resource and Environment Data Cloud Platform (http://www.resdc.cn). The vegetation types in northeast China were summarized as follows: coniferous and broadleaf mixed forest (CBMF), broadleaf forest (BF), coniferous forest (CF), herbosa (HE), shrubland (SH), swamp (SW), meadow (ME), cropland (CR), grassland (GR), and others (OT, such as water area). Soil included the following 23 types: brown coniferous forest soil (BCFS), brown forest soil (BFS), dark-brown forest soil (DBFS), albic soil (AS), cinnamon soil (CS), phaeozem (PH), gray forest soil (GFS), chernozem (CH), chestnut soil (CHS), chestnut cinnamon soil (CCS), alluvial soil (ALS), aeolian sandy soil (ASS), andisol (AN), lithosol (LI), skeletal soil (SS), meadow soil (MS), fluvo-aquic soil (FAS), bog soil (BOS), peat soil (PS), solonchak (SO), marine solonchak (MAS), solonetz (SOL,) and paddy soil (PAS). Vegetation type and soil type data (with a scale of 1:1,000,000) were resampled to 0.25° by nearest-neighbor interpolation. 

Daily precipitation for 2007–2015 at a 0.5° spatial resolution was obtained from the National Meteorological Information Center of China (http://data.cma.cn/). The gridded datasets were produced through the thin-plate spline (TSP) interpolation method in ANUSPLIN software using the meteorological data from 2472 national meteorological stations across China. We applied the MVC method to generate eight-day precipitation data.

### 2.3. Methods

#### 2.3.1. Calculation of Soil Water Use Efficiency (SWUE)

SWUE describes the rate of carbon assimilation per unit of soil water loss, which links the photosynthetic productivity of ecosystems to soil water. In this study, the eight-day SWUE of each pixel in northeast China was calculated by soil moisture (SM) and gross primary productivity (GPP), expressed by the following formula:

(1) $$\mathrm{SWUE}\;=\;\frac{\mathrm{GPP}}{\mathrm{SM}}.$$

The units of GPP, SM, and SWUE were gC/m^2^, m^3^∙m^−3^, and gC/kg H_2_O, respectively.

#### 2.3.2. Spatial Change Trend Analysis

The coefficient of variation (CV) can measure the dispersion degree of data. In this study, we calculated CV of each pixel’s SWUE from 2007 to 2015 to characterize the spatial distribution and variations of ecosystem surface SWUE in northeast China. The formulas are as follows:

(2) $$\mathrm{CV}=\frac{\mathrm{SD}}{\bar{\mathrm{SWUE}}},\;\mathrm{SD}\;=\;\sqrt{\frac{1}{n}\sum_{i=1}^{n}{{(\mathrm{SWUE}}_{i}-\bar{\mathrm{SWUE}})}^{2}},\;\bar{\mathrm{SWUE}}=\frac{1}{n}\sum_{n=1}^{n}{\mathrm{SWUE}}_{i}.$$

Spatial trends of SWUE were explored using a linear regression model with time as the independent variable and SWUE as the dependent variable [34]. The slope of the fitted regression line at each pixel was calculated using Equation (3).

(3) $$\mathrm{slope}=\frac{n\times \sum_{i=1}^{n}i\times {\mathrm{SWUE}}_{i}-\sum_{i=1}^{n}i\sum_{i=1}^{n}{\mathrm{SWUE}}_{i}}{n\times \sum_{i=1}^{n}i^{2}-{\left(\sum_{i=1}^{n}i\right)}^{2}},$$

where *n* represents the number of years, SWUE*_i_* is the summed SWUE in growing season for year *i*, $\bar{\mathrm{SWUE}}$ represents average SWUE during *n* years, and SD is the standard deviation. As the CV becomes smaller, the value of SWUE becomes more stable. The *F*-statistic was used to determine the significance of the linear regression model. Based on the slope value and significant levels, the change trends were classified into five grades: extremely significant increase (slope > 0, 0 < *p* ≤ 0.05), significant increase (slope > 0, 0.05 < *p* ≤ 0.1), no significant change (*p* > 0.1), significant decrease (slope < 0, 0.05 < *p* ≤ 0.1), and extremely significant decrease (slope < 0, 0 < *p* ≤ 0.05).

#### 2.3.3. Phenological Metrics Extraction

The phenological metrics were extracted from MOD13A2 NDVI data. Zhao et al. reported that there was little difference among Gaussian, logistic, and Savitzky–Golay fitting methods in the TIMESAT tool [23]. We used TIMESAT software in MATLAB R2015b (The Mathworks, Inc., Natick, MA, USA) with a seasonal parameter of 0.5, an adaptation strength of 2.0, a Savitzky–Golay window size of 2, and an amplitude of 20% to calculate the start of season (SOS), the end of season (EOS), and the length of season (LOS) in northeast China from 2007 to 2015.

#### 2.3.4. Correlation Analysis

Pearson correlation is regarded as a standard method to analyze the relationship between two variables. To examine the response of SWUE variations to vegetation activity, land surface temperature, and precipitation dynamics, the correlation coefficients between eight-day time series SWUE and NDVI, LST, and precipitation were calculated for each pixel, respectively. To investigate the role of photosynthetic phenological factors affecting SWUE, we analyzed the Spearman partial correlations between the accumulated SWUE in the growing season and the SOS and EOS. The equations of the correlation coefficient and partial correlation coefficient were as follows:

(4) $$r_{\mathrm{xy}}=\frac{\sum_{i=1}^{n}\left(x_{i}-\bar{x}\right)\left(y_{i}-\bar{y}\right)}{\sqrt{\sum_{i=1}^{n}{\left(x_{i}-\bar{x}\right)}^{2}}\sqrt{\sum_{i=1}^{n}{\left(y_{i}-\bar{y}\right)}^{2}}},$$

(5) $$\bar{x}=\frac{1}{n}\sum_{i=1}^{n}x_{i},\;\bar{y}=\frac{1}{n}\sum_{i=1}^{n}y_{i},$$

(6) $$r_{\mathrm{xy},z}=\frac{r_{\mathrm{xy}}-r_{\mathrm{xz}}r_{\mathrm{yz}}}{\sqrt{1-r_{\mathrm{xz}}^{2}}\sqrt{1-r_{\mathrm{yz}}^{2}}},$$

where r_xy_ represents the correlation coefficient between *x* and *y*, ranging from −1 to 1, and r_xy,z_ means the partial correlation coefficient between *x* and *y* when we controlled *z* values. If r < 0, *x* is negatively correlated with *y*. If r > 0, there is a positive correlation between *x* and *y*. Furthermore, $\bar{x}$, $\bar{y}$ represent the mean values of *x*_i_ and *y*_i_, respectively. The absolute value of r indicates the relevance of two variables. The greater the value of |r| is_,_ the closer the two variables are and vice versa. In generally, 0 < |r| < 0.3 indicates a weak correlation between two variables, while 0.3 ≤ |r| < 0.6 means moderate correlation, and strong correlation exists in two variables when 0.6 ≤ |r| < 1. The significance of the results was examined using a *t*-test. 

## 3. Results

### 3.1. Spatial Distribution Patterns of SWUE

**Figure 2:**
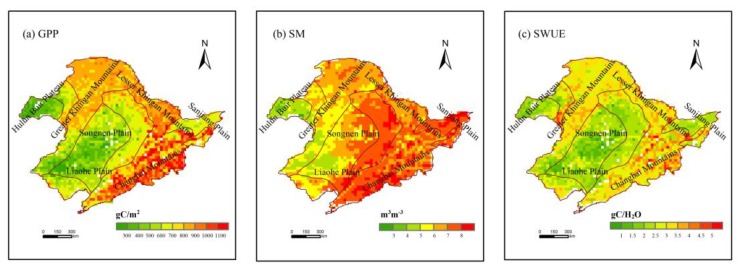
The multi-year average gross primary productivity (GPP) (**a**), soil moisture (SM) (**b**), and soil water use efficiency (SWUE) (**c**) in the growing season of northeast China from 2007 to 2015.

The multi-year average GPP, SM, and SWUE data during the growing season in northeast China from 2007 to 2015 are shown in Figure 2. The spatial distribution of GPP showed significant heterogeneity (Figure 2a). In the Songnen Plain, Liaohe Plain, and Hulun Buir Plateau, GPP values of cropland and grassland were lower. The lowest value was about 94.3047 gC/m^2^. The GPP values of vegetation in Changbai Mountain and the Greater and Lesser Khingan Mountains were relatively high with the maximum value of 1343.26 gC/m^2^. The multi-year average surface SM in northeast China was 3.001–9.431 m^3^∙m^−3^. Similar characteristics of SM spatial distribution to that of GPP were found (Figure 2b). The SM values were lower in the west and southwest of northeast China. Relatively high SM was observed in the middle, east, and southeast of northeast China and Sanjiang Plain wetland. The multi-year average SWUE ranged from 0.341 gC/kg H_2_O to 6.173 gC/kg H_2_O (Figure 2c), showing consistent spatial distribution of GPP. Low SWUE values were found in the middle and southwest, whereas SWUE in the east and southeast of the study area was higher.

**Figure 3:**
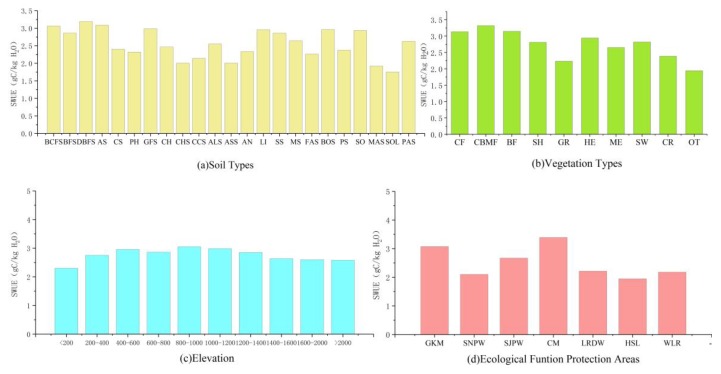
The variations of multi-year average soil water use efficiency (SWUE) in growing season with soil types (**a**), vegetation types (**b**), elevation (**c**), and ecological function protection areas (**d**) in northeast China.

Ecosystem SWUE varied in different soil types (Figure 3a). SWUE values on dark-brown forest soils were the highest (3.187 gC/kg H_2_O), followed by albic soils (3.084 gC/kg H_2_O). The lowest SWUE (1.752 gC/kg H_2_O) was observed in solonetz, and marine solonchak showed an average SWUE of 1.920 gC/kg H_2_O. During the past nine years, the order of growing season SWUE of different vegetation in northeast China was as follows: CBMF > BF > CF > HE > SH > SW > ME > CR > GR > OT. Highest SWUE was observed in coniferous and broadleaf mixed forests (3.319 gC/kg H_2_O), followed by broadleaf forests (3.314 gC/kg H_2_O). The lowest SWUE was found in area covered by other type (1.942 gC/kg H_2_O) (Figure 3b). SWUE values for grasslands were relatively lower (2.231 gC/kg H_2_O). SWUE also varied with the elevation. Highest multi-year average SWUE was found at an elevation of 800–1000 m (3.051 gC/kg H_2_O). The average SWUE values in the areas of below 200 m and above 2000 m were 2.301 gC/kg H_2_O and 2.583 gC/kg H_2_O, respectively. From 0 to 1000 m, SWUE increased gradually. When the elevation was over 1000 m, there was a decreasing trend in SWUE (Figure 3c).

As shown in Figure 3d, SWUE of vegetation was generally high in the ecological function protection areas for water conservation in northeast China. Average SWUE values in the Changbai Mountains and the Great Khingan Mountains were 3.388 gC/kg H_2_O and 3.073 gC/kg H_2_O, respectively. The Sanjiang and Songnen Plain wetland ecological function protection areas play important roles in flood regulation, with average SWUE values of 2.669 gC/kg H_2_O and 2.100 gC/kg H_2_O, respectively. As the only ecological function protection area of species resources in northeast China, average SWUE in the Liaohe River Delta Wetlands reached 2.179 gC/kg H_2_O. In Horqin Sandy Land, the ability and role of ecosystems to prevent land desertification and reduce the risk of sandstorm is mainly emphasized. However, vegetation SWUE in this area was the lowest (1.945 gC/kg H_2_O).

### 3.2. Temporal Variation of SWUE 

During 2007–2015, ecosystem SWUE in northeast China showed fluctuating changes. The multi-year average SWUE peaked in 2007 (2.925 gC/kg H_2_O) and then decreased. In 2009, SWUE fell to 2.65 gC/kg H_2_O, while SWUE continued to increase in 2010–2012. In 2013, SWUE dropped to the lowest value (2.550 gC/kg H_2_O). Regional SWUE increased in the following two years and reached 2.78 gC/kg H_2_O in 2015.

**Figure 4:**
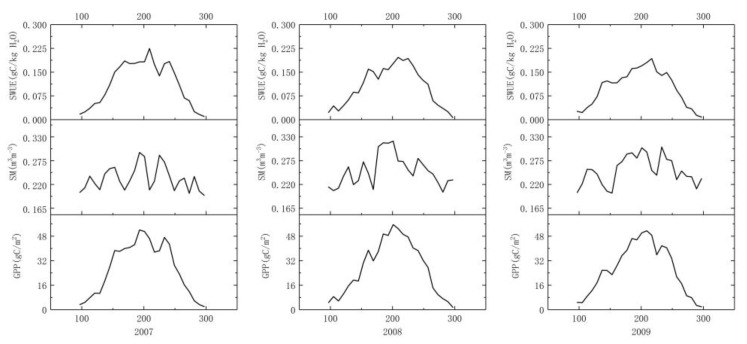

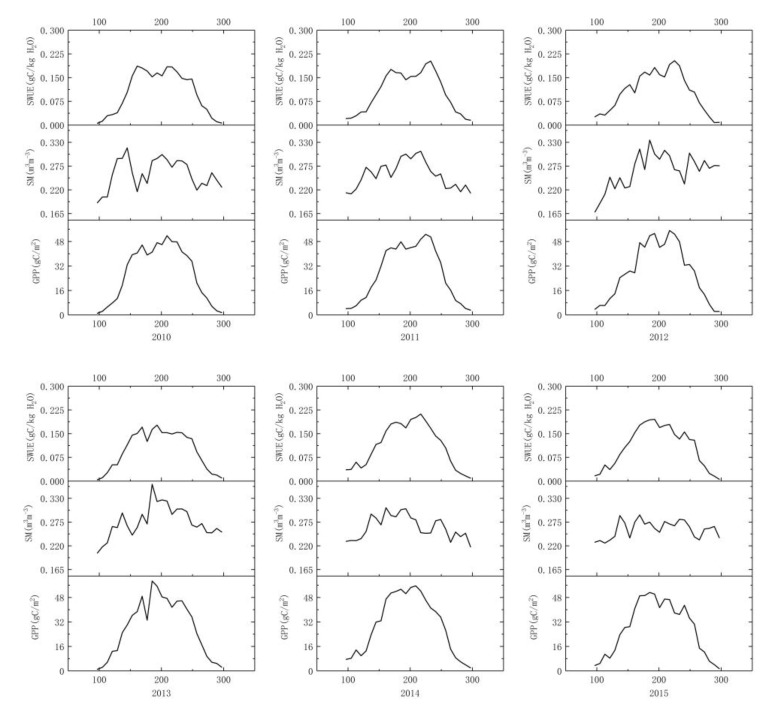
The variations of eight-day soil water use efficiency (SWUE), soil moisture (SM), and gross primary productivity (GPP) in northeast China from 2007 to 2015.

Figure 4 shows the variations of eight-day SWUE, SM, and GPP in the growing season from 2007 to 2015. In general, SWUE increased continuously from spring to summer (from April to early August) and gradually decreased from the middle of August to October, which was consistent with the trend of GPP variation in northeast China. Except for the years of 2010, 2013, and 2015, the highest SWUE values mainly occurred from the end of July to August. At the end of July 2007 (day 209), SWUE was the highest (0.225 gC/kg H_2_O), followed by that in 2014 (0.212 gC/kg H_2_O, day 217). Lowest SWUE values in 2010 and 2013 were found in early April (day 97), while they were observed at the end of October (day 297) for other years. On the 97th day of 2013, SWUE was the lowest (0.004 gC/kg H_2_O). The highest multi-year average SWUE was 0.181 gC/kg H_2_O on the 217th day. By contrast, the lowest multi-year average value of vegetation SWUE was 0.008 gC/kg H_2_O on day 297.

### 3.3. Spatial Change Trends of SWUE 

**Figure 5:**
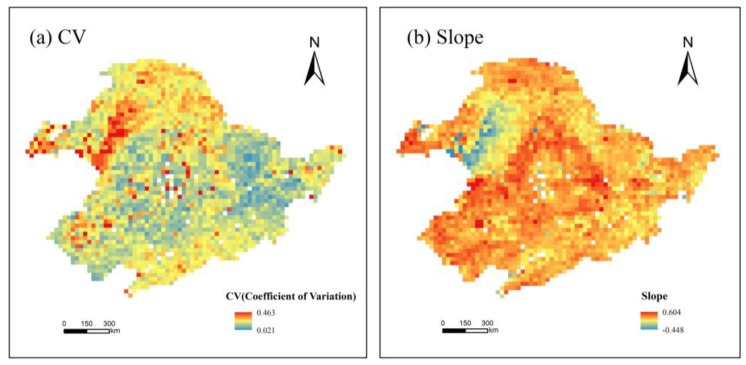
The coefficient of variation (**a**) and slope (**b**) of soil water use efficiency (SWUE) in northeast China from 2007 to 2015.

Figure 5a illustrates the spatial distribution of the CV of SWUE. The CV values greater than 0.2 accounted for 0.9% of the studied area, and CV ranged from 0 to 0.2 in the remaining areas. From Figure 5b, the slope values of SWUE were negative in 49% of the total land, whereas positive slopes were found in 51% of the study area. Spatially, there were no significant variation trends of SWUE (*p* > 0.2). The results of the significance test showed that the accumulated SWUE in the growing season was relatively stable in northeast China for the period of 2007–2015.

### 3.4. Effects of NDVI, LST, and Precipitation Changes on SWUE Variability

**Figure 6:**
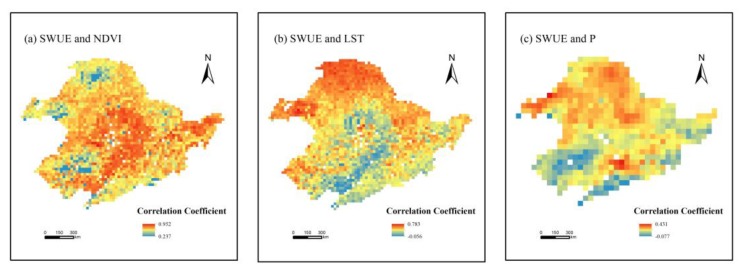
The spatial patterns of correlation coefficients between soil water use efficiency (SWUE) and NDVI (**a**), land surface temperature (LST) (**b**), and precipitation (**c**).

To reveal the response of SWUE changes to vegetation dynamics in northeast China, we calculated the correlation coefficients between SWUE and NDVI in the period of 2007–2015 (Figure 6a). We found that 98.7% of the study area showed strong correlation (0.6 ≤ *r* < 1, *p* < 0.05), while weak to moderate correlation was observed in 1.3% of the landscape. SWUE showed a strong and positive correlation with NDVI. In the region with greater NDVI, which indicated enhanced vegetation activity, the SWUE of vegetation might also have improved.

In Figure 6b, the correlation between SWUE and LST in northeast China from 2007 to 2015 is presented. SWUE was highly and moderately correlated with LST, accounting for 19.0% and 63.1% of the land that passed the significance test (taking up 96.8% of the study area, *p* < 0.05), respectively. The area showing weak correlation occupied only 17.9% of the study area. Overall, LST in the growing season had positive effects on SWUE variations in the study area. As LST increased, SWUE might have improved.

As a supply of soil moisture, precipitation affects vegetation growth and the maintenance of ecosystem services, especially in arid and semi-arid areas. We resampled the time-series SWUE data to a 0.5° spatial resolution to match the precipitation data, using the bilinear interpolation method. The correlation coefficient between eight-day SWUE and precipitation in the growing season was then calculated (Figure 6c). It was found that SWUE was significantly positive correlated with precipitation in 84% of the study area (*r* > 0, *p* < 0.05), with 14.2% and 85.8% of the area presenting moderate and weak correlation, respectively. In the last nine years, the vegetation SWUE was positively correlated with the precipitation in northeast China, although the correlation was not strong. With the increase of precipitation, the value of SWUE increased.

### 3.5. Response of SWUE to Phenological Variation

#### 3.5.1. Spatial Distribution of SOS, LOS, and EOS

**Figure 7:**
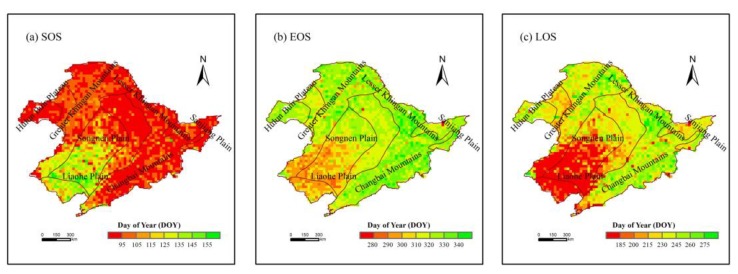
The average start (SOS) (**a**), end (EOS) (**b**), and length (LOS) (**c**) of the growing season in northeast China from 2007 to 2015.

Figure 7 shows the pixel-scale spatial distribution of land surface phenological parameters (SOS, EOS, and LOS) in northeast China from 2007 to 2015. It was found that the average SOS mainly occurred between May and early June (120–155th day of the year (DOY)) in the middle of the Songnen Plain and the Liaohe Plain. In the Changbai Mountains, Greater Khingan Mountains, and Lesser Khingan Mountains, vegetation had early SOS dates (95–120th DOY, mainly in April). The spatial distribution of EOS dates was significantly different from that of SOS and gradually became later from south to north. In the Greater Khingan Mountains, Lesser Khingan Mountains, Changbai Mountains, and the north of the Songnen Plain, the average EOS dates were mainly between the end of October and November (300–340th DOY). However, the EOS of vegetation in the central and southern part of Songnen Plain and Liaohe Plain was early in October (280–300th DOY). During the past nine years, the average LOS was 223 days in northeast China, with a similar spatial distribution to that of EOS. The LOS (<200 days) was observed in the cropland of the south of Songnen Plain and Liaohe Plain, while the LOS in other areas was mostly between 200 and 275 days.

#### 3.5.2. Partial Correlations between SWUE and Phenological Metrics

**Figure 8:**
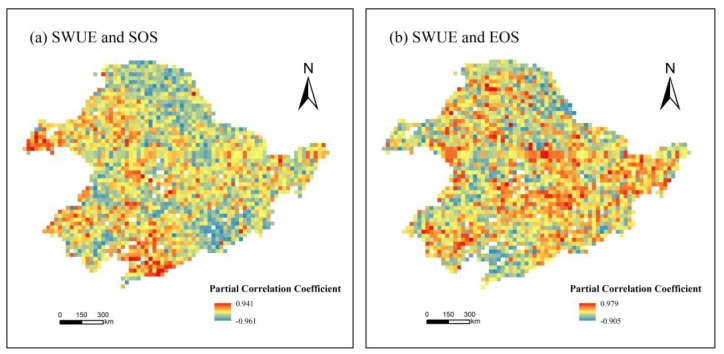
The relationship between soil water use efficiency (SWUE) and the start (SOS) (**a**) and end (EOS) (**b**) of the growing season.

Figure 8a shows the spatial distribution of partial correlation coefficients between SWUE and SOS in the growing season. In 59.3% of the study area, *r* was less than 0, while 40.7% of the area showed positive partial correlation coefficients. Overall, there existed significant correlation between SOS and SWUE in 2.4% of the total area (*p* < 0.05). In these areas, the proportions of pixels that were negatively or positively correlated with SWUE were 67.2% (*r* < 0) and 32.8% (*r* > 0), respectively. It may be concluded that vegetation SWUE was higher with earlier SOS in northeast China during the last nine years.

From Figure 8b, in 51.8% of the study area, SWUE was positively associated with EOS, while a negative correlation between SWUE and EOS was found in the remaining area (taking up 48.2% of the total land). About 1.9% of the study area passed the significance test (*p* < 0.05). The areas showing positive and negative correlations between EOS and SWUE accounted for 73.3% and 26.7%, respectively. Thus, the later EOS is, the greater the SWUE of vegetation could be.

## 4. Discussion

### 4.1. Spatial and Temporal Characteristics of SWUE in the Growing Season

The average growing season SWUE in northeast China illustrated a clear spatial heterogeneity during the past nine years. SWUE ranged between 0.341 and 6.173 gC/kg H_2_O. The SWUE values of cropland and grassland in the Songnen Plain, Liaohe Plain, and Hulun Buir Plateau were relatively lower compared to the regional average. According to our statistics, the GPP values of cropland and grassland were relatively low. The SM in grassland was lowest, but SM was higher in cropland. The lower SWUE values were possibly due to more consumption of soil water by soil evaporation in the ecosystems. Relatively high SWUE was observed in the Changbai Mountains, Greater Khingan Mountains, and Lesser Khingan Mountains, indicating that forests might produce greater photosynthetic productivity per unit of soil water consumption. Xiao et al. also found that WUE was higher in forest, and that the WUE of grassland and cropland was relatively low in China using site data [3].

During the growing season from 2007 to 2015, SWUE fluctuated to a certain extent in northeast China. SWUE increased continuously from April to August, suggesting better efficiency of ecosystems in assimilating carbon by soil water consumption in spring and summer. The lowest SWUE mainly appeared at the end of October, showing the weak capability of photosynthetic productivity of the ecosystem generated by the use of limited soil water supply in autumn. This trend was consistent with the vegetation growth cycle. Note that SWUE variations in northeast China were more consistent with GPP than soil moisture, because the range of annual cumulative GPP (96.304–1343.26 gC/m^2^) was larger than that of SM (3.001–9.431 m^3^∙m^−3^). The interannual variation of SWUE was generally stable in northeast China from 2007 to 2015. Spatially, SWUE exhibited insignificantly change trends, possibly because our study period was relatively short. He et al. also found that the distribution of SWUE was spatially heterogeneous at a global scale, but the interannual variations of SWUE were not illustrated in their study [9]. 

### 4.2. Relationship between SWUE, Climate, Geography, and Phenology Factors

To date, few studies on SWUE and its driving forces were conducted. Similar to WUE, SWUE reflects the coupling of the carbon–water cycle of terrestrial ecosystems and may be affected by environmental and biotic factors such as climate factors, soil features, and phenological factors [9]. In this current study, we found that the growing season SWUE tended to increase with the increase of NDVI, LST, and precipitation in northeast China. Since April, vegetation gradually developed from seedling to maturity, while NDVI and LST values kept increasing, and precipitation also increased. Good hydrothermal conditions during the summer were favorable for vegetation growth. Some previous studies indicated that, when leaf area of vegetation becomes larger, vegetation will reduce the loss of soil moisture by preventing evaporation, thereby potentially increasing SWUE [9,35]. The SWUE showed relatively weak and positive correlations with precipitation in the study area, which was related to the decreasing water supply from precipitation due to the surface runoff. Some similar researches also showed that high WUE is related to high temperature, high precipitation, and high NDVI [3,36].

At the biome level, SWUE showed large differences. In our study, higher SWUE in the growing season was observed in coniferous and broadleaf mixed forests, broadleaf forests, and coniferous forests in northeast China. The cold temperate coniferous forests are mainly distributed in the north of the Great Khingan Mountains, including evergreen or deciduous coniferous forests with frost resistance. Although there is lower precipitation and evaporation, the coniferous forests with cold and drought tolerance can grow better than other vegetation. The warm temperate deciduous broadleaf forests are mainly distributed in the south of Liaoning Province. The coniferous and broadleaf mixed forests are composed of evergreen coniferous, deciduous coniferous, and deciduous broadleaf forests, which are distributed in the south of the Great Khingan Mountains and the Lesser Khingan Mountains and the Changbai Mountains. The wide leaf area of broadleaf forest could reduce soil moisture loss; thus, SWUE was relatively high. He et al. found that evergreen broadleaf forests had the highest mean annual SWUE, while shrubs had the lowest value at a global scale for the period of 2000–2014 [9]. Both findings suggested that forests could generate higher productivity per unit of soil water consumption. The differences in SWUE values might be because the researches were conducted at a global scale and regional scale, respectively, and the classification schemes of forests were somewhat different.

The SWUE distribution varied among soil types. Coniferous and broadleaf mixed forests are mainly distributed on dark-brown forest soils, where the highest SWUE values in the growing season were observed. Lowest SWUE was identified for solonetz in arid and semi-arid areas in northeast China, which was in agreement with He et al.’s result of lower annual SWUE in arid ecosystems. Topography directly affects the material flow and energy transformation of the land surface. During the study period, vegetation SWUE showed a certain regularity with elevation change in northeast China. Below 1000 m, SWUE presented a rising trend. The cropland was mainly distributed below 400 m, where SWUE was marginally associated with the greater anthropogenic influence. Coniferous forests and broadleaf forests were mainly distributed between 800 and 1000 m, where vegetation SWUE reached the maximum. When the elevation was more than 1000 m, the SWUE decreased gradually as elevation increased. This was related to the decrease of GPP in vegetation with the elevation increasing.

The variations of growing season length have important effects on vegetation growth. The results of this study show that SWUE increased with earlier SOS and later EOS in northeast China. Similarly, Jin et al. found that WUE is correlated to SOS and EOS, whereby the increase of WUE also occurred with earlier SOS and later EOS [18]. Xiao et al.’s study indicated that the growing season length would affect the annual WUE [3]. Early SOS might make the growing season longer and result in the accumulation of GPP increase. The germination and growth of leaves could be promoted [37]. Leaf area increased gradually, and the photosynthesis of vegetation was enhanced. In spring, soil evaporation was not very large due to temperature restriction, and increasing vegetation coverage could prevent the evaporation of soil moisture [38]. Thus, SWUE increased with early SOS. Soil water deficit often occurred in autumn, and later EOS also led to the increase in growing season. Therefore, with the accumulation of GPP increasing, soil water use efficiency of the vegetation was higher.

### 4.3. Uncertainty

It should be noted that the lower spatial resolution of the available soil moisture product (0.25°) unavoidably affected the accuracy of SWUE, especially for highly heterogeneous ecosystems. The ECV-SM data only represented the surface soil moisture condition [9]; thus, the estimation of SWUE might ignore deeper soil water used by plants. In addition, ECV-SM products cannot accurately capture the soil moisture dynamics in dense vegetation areas, which limits the reliability of SWUE. There still exists the problem of missing data in the CCI soil moisture product. In this study, due to the lack of CCI soil moisture data, especially before 2007, we could not obtain and analyze longer time-series SWUE in northeast China. NDVI, LST, precipitation, and land surface phenology parameters were chosen as the representative factors when evaluating the relationship among SWUE, and environmental and phenological changes. Some other variables such as soil evaporation and irrigated water supplies may also be associated with the ecosystem SWUE. Moreover, the correlation analyses between SWUE and the above impact factors were preliminary and better-fitting mathematical methods should be explored in further study. 

In addition, the land surface phenology results varied because different data sources and models were adopted. In many previous studies, the analysis of LSP would not include the cropland because it was highly affected by human activity. Due to the large area of cropland and the low resolution of estimated SWUE data, this study was conducted on the whole region including cropland. The phenological parameters were compared with the studies of Zhao et al. [23] (used Global Inventory Modeling and Mapping Studies (GIMMS) NDVI3g data), Wang et al. [39] (used Advanced Very High Resolution Radiomete (AVHRR) NDVI data), and Luo et al. [40] (Table 1). The deviations may be attributed to human interference and variations in natural conditions. The results of this study were reasonable. 

## 5. Conclusions

Soil moisture can regulate vegetation productivity and affect terrestrial carbon uptake. Soil moisture-based water use efficiency may promote the understanding of soil water use in various ecosystems and ecological functions. This study investigated spatial and temporal patterns of SWUE in northeast China, integrating time series of MODIS GPP products and daily soil moisture data (ECV-SM) derived from microwave sensors. The spatial distribution of average SWUE was consistent with that of GPP. We identified that the variations of SWUE in northeast China were obvious within a year. There was an increasing trend of SWUE between April and August, but SWUE decreased gradually from August to October. The annual cumulative SWUE showed insignificant change trends from 2007 to 2015. The SWUE values showed appreciable differences among different elevations, biomes, soil types, and ecological function protection areas. Above 1000 meters, SWUE decreased with elevation. Highest SWUE values were observed in coniferous and broadleaf mixed forests (3.319 gC/kg H_2_O), dark-brown forest soils (3.187 gC/kg H_2_O), and the ecological function zone of the Changbai Mountains (3.388 gC/kg H_2_O). SWUE was positively correlated with NDVI, LST, and precipitation. The earlier SOS and later EOS exerted a positive influence on SWUE in northeast China. Phenology may play a role in the regulation of terrestrial ecosystem soil water use efficiency.

## Figures and Tables

**Table 1:** Comparisons of calculated land surface phenology (LSP) metrics and existing research results.

Study Area	SOS/DOY	EOS/DOY	Study Period	Data	Source
Northeast China	95–155	280–340	2007–2015	MODIS MOD13A2	This study
Northeast China	115–155	300–340	1982–2013	GIMMS NDVI3g	Zhao [23]
North China	80–190	260–310	1982–1999	AVHRR NDVI	Wang [39]
China	50–170	225–345	2001–2014	MODIS MOD13A2	Luo [40]
